# LIFE PROBLEMS, PROVIDING ADVICE, AND DAILY MOOD IN LATE LIFE

**DOI:** 10.1093/geroni/igad104.2191

**Published:** 2023-12-21

**Authors:** Sibo Gao, Shiyang Zhang, Karen Fingerman

**Affiliations:** The University of Texas at Austin, Austin, Texas, United States; The University of Texas at Austin, Austin, Texas, United States; The University of Texas at Austin, Austin, Texas, United States

## Abstract

Older adults often experience life problems (e.g., health conditions, financial crises) that may be detrimental to their well-being. Yet, meaningful social connections and support exchanges may mitigate the negative effect of life problems on older adults’ emotional health. This study used data from the Daily Experiences and Well-being Study to investigate how providing and receiving advice influence the association between life problems and mood. Participants aged 65+ (Mage = 74.15, N = 293) completed an initial interview in which they indicated whether they had different types of life problems (health, financial, legal). Participants then completed an ecological momentary assessment in which they reported their negative and positive mood every 3 hours for 5 to 6 consecutive days. They also reported whether they provided or received advice from any social partner at the end of each day. Results showed that participants with more life problems reported higher negative mood throughout the day. Multilevel models revealed a significant interaction between life problems and providing advice on mood, such that the number of life problems was positively associated with negative mood on days that participants did not give advice, whereas life problems were not associated with negative mood on days that participants gave advice. Receiving advice did not reduce the adverse effect of life problems on negative mood. Findings underline that providing (but not receiving) advice may promote generativity among older adults and thus benefit their well-being. The study provides important implications for improving older adults’ well-being especially when they face life problems.

